# Extracellular vesicles from human mesenchymal stem cells expedite chondrogenesis in 3D human degenerative disc cell cultures

**DOI:** 10.1186/s13287-020-01832-2

**Published:** 2020-07-29

**Authors:** Daphne Hingert, Karin Ekström, Jonathan Aldridge, Rosella Crescitelli, Helena Brisby

**Affiliations:** 1 Department of Orthopedics, Institute of Clinical Sciences, Sahlgrenska Academy, University of Gothenburg, Gothenburg, Sweden; 2 Sahlgrenska Cancer Center, Department of Surgery, Institute of Clinical Sciences, Sahlgrenska Academy, University of Gothenburg, Gothenburg, Sweden; 3 Wallenberg Centre for Molecular and Translational Medicine, University of Gothenburg, Gothenburg, Sweden; 4 Department of Rheumatology and Inflammation Research, Institute of Medicine, University of Gothenburg, Gothenburg, Sweden; 5 Department of Orthopedics, Sahlgrenska University Hospital, Gothenburg, Sweden

**Keywords:** Extracellular vesicles, Exosomes, Human mesenchymal stem cells, Intervertebral disc cells, Low back pain, Chondrogenesis

## Abstract

**Background:**

Extracellular vesicles (EVs) from human mesenchymal stem cells (hMSCs) are known to be mediators of intercellular communication and have been suggested as possible therapeutic agents in many diseases. Their potential use in intervertebral disc (IVD) degeneration associated with low back pain (LBP) is yet to be explored. Since LBP affects more than 85% of the western population resulting in high socioeconomic consequences, there is a demand for exploring new and possibly mini-invasive treatment alternatives. In this study, the effect of hMSC-derived small EVs (sEVs) on degenerated disc cells (DCs) isolated from patients with degenerative discs and chronic LBP was investigated in a 3D in vitro model.

**Methods:**

hMSCs were isolated from bone marrow aspirate, and EVs were isolated from conditioned media of the hMSCs by differential centrifugation and filtration. 3D pellet cultures of DCs were stimulated with the sEVs at 5 × 10^10^ vesicles/ml concentration for 28 days and compared to control. The pellets were harvested at days 7, 14, and 28 and evaluated for cell proliferation, viability, ECM production, apoptotic activity, chondrogenesis, and cytokine secretions.

**Results:**

The findings demonstrated that treatment with sEVs from hMSCs resulted in more than 50% increase in cell proliferation and decrease in cellular apoptosis in degenerated DCs from this patient group. ECM production was also observed as early as in day 7 and was more than three times higher in the sEV-treated DC pellets compared to control cultures. Further, sEV treatment suppressed secretion of MMP-1 in the DCs.

**Conclusion:**

hMSC-derived sEVs improved cell viability and expedited chondrogenesis in DCs from degenerated IVDs. These findings open up for new tissue regeneration treatment strategies to be developed for degenerative disorders of the spine.

## Background

Low back pain (LBP) affects more than 85% of the western population resulting in high socioeconomic consequences [1]. Chronic LBP is caused by multifactorial pathologies and further affected by psychosocial factors [2]. A certain interest has been directed towards intervertebral disc (IVD) degeneration, believed to be one of the major underlying pathologies [3]. It involves loss of proteoglycan and extracellular matrix (ECM) and increased expressions of pro-inflammatory cytokine and matrix metalloproteinases (MMPs), which affects the viability of the disc cells (DCs) in the IVDs [4, 5]. Current treatment options are symptomatic and do not address the underlying degenerative process or promote regeneration. New treatment strategies with effective molecular agents that can be clinically applied in minimally invasive therapies are therefore warranted. Mesenchymal stem cell (MSC)-based therapies are of interest in tissue repair and regeneration due to their multilineage differentiation and immunomodulatory abilities [6]. One suggested strategy is injection of MSCs into degenerated IVDs and/or incorporation of growth factors to boost differentiation [7, 8]. However, growing evidence from studies on MSC effects on disc cells suggests that MSCs exert their therapeutic effects through paracrine signaling by secretion of bioactive factors and soluble peptides [9]. Extracellular vesicles (EVs) are secreted by the MSCs as mediators of intercellular communication, and they have shown a potential to drive regenerative processes in many diseases [10–12]. EVs have been reported to inhibit apoptosis in nuclear pulposus (NP) cells and suppress IVD degeneration in rat-tail models [13].

This study aimed to investigate the potency of small EVs (sEVs) isolated from hMSCs on degenerated DCs obtained from patients with degenerated IVDs and chronic LBP in a 3D in vitro model. The evaluation of the experiments was carried out in terms of cell proliferation, viability, ECM production, apoptotic activity, chondrogenesis, and cytokine secretions with the ambition that these findings, which are explicitly in connection with patients’ cells, would pave ways for development of new regeneration strategies for treatments of spine degenerative disorders.

## Methods

### Patients

Intervertebral disc (IVD) tissues and bone marrow aspirates (BMAs) from iliac crest were collected from patients with chronic low back pain diagnosed with IVD degeneration undergoing spinal fusion surgery including removal of the intervertebral disc(s). The IVDs were graded according to the Pfirrmann grading system on magnetic resonance imaging as grade 3–4. Disc tissues collected were obtained from the center of the IVD, aiming for NP tissue. However, due to disintegration of the IVD tissues, the collected tissues were considered a mix of NP and annulus fibrosus tissues. IVD tissues were obtained from 4 females and 1 male donors, age 34–50, while BMA was obtained from 1 female donor, age 40.

### Human MSC isolation and culture

Human mesenchymal stem cells (hMSCs) were isolated and cultured as previously described in [8]. Briefly, BMA was collected in 3.2% sodium citrate (Greiner Bio One, Kremsmuenster, Austria) after surgery and then centrifuged (470×*g*, 20 min). The mono-nuclear phase was collected and cultured in culture flasks (Corning, NY, USA) (37 °C and 5% CO_2_) in MSC media (DMEM-low glucose and 110 mg/l sodium pyruvate, with 4 mmol/l l-glutamine (Thermo Fisher Scientific, MA, USA) and 1% penicillin/streptomycin, 4 ng/ml beta-fibroblast growth factor (FGF)) (Thermo Fisher Scientific) and 10% human serum (HSE) (isolated in-house from donor’s blood obtained from GeBlod, Gothenburg). Media were changed every 2 days, and the cells were passaged at 90% confluency.

### Disc cell isolation and culture

Disc tissues were cut into small pieces and transferred to culture flasks (Corning, NY, USA) with an addition of 1 mg/ml type II collagenase (Gibco Life, MA, USA). After a 24-h incubation (37 °C and 5% CO_2_), the cell suspension was centrifuged (470×*g*, 4 °C, 5 min) and the cells were cultured (37 °C and 5% CO_2_) in DC media (DMEM-low glucose with 110 mg/l sodium pyruvate, 4 mmol/l l-glutamine, 1% penicillin/streptomycin (Thermo Fisher Scientific), and 10% HSE). The media were changed every 2 days, and the disc cells (DCs) were passaged at 90% confluency. All the DCs used in the study were in passage 5–6.

### Human MSC characterization

The hMSCs were analyzed to confirm the expressions of CD73, CD90, and CD105 and the absence of CD11b, CD19, CD34, CD45, and HLA/DR using BD Stemflow hMSC analysis kit (BD Biosciences, CA, USA). The samples were prepared according to the manufacturer’s instructions, and the data were acquired using a BD FACSVerse™ instrument (BD Biosciences) and analyzed using FlowJo™10 (Flow Jo, LLC, Ashland, OR, USA).

### Conditioned media collection and EV isolation

hMSCs (approximately 10,000 cells/cm^2^) were seeded in MSC media containing human serum for 24 h to allow cell attachment to the bottom of the flasks. The MSC media were removed, and the culture flask washed twice with phosphate buffer saline (PBS) prior to adding serum-free media (MSC NutriStem XF medium, Biological Industries, Kibbutz Beit-Haemek, Israel). Conditioned media (CM) were collected every 48 h, and the cells were passaged at 90% confluency. CM collected were centrifuged (300×*g*, 4 °C, 5 min) and stored at − 80 °C until use. The hMSCs used in the study were in passage 4–7. The isolation of extracellular vesicles (EVs) was performed by a series of differential centrifugation and filtration of the CM as described in [14]. Briefly, CM were thawed on water bath and centrifuged at 16,500×*g* for 20 min followed by filtration through 0.22-μm filters to deplete cell debris and large EVs. The sEV/exosomes were then pelleted by ultracentrifugation at 120,000×*g* for 70 min in a T-645.5 rotor (Sorvall wx Ultra series, Thermo Scientific, Rockford, IL, USA). The sEV pellets were re-suspended in cold PBS and stored at − 80 °C until use. The whole procedure was performed at 4 °C.

### Characterization of EVs

#### Nanoparticle tracking analysis

The concentration and size distribution of the sEVs were determined by nanoparticle tracking (NTA). Briefly, the sEV samples were diluted (200× and 1000×) with PBS and analyzed with Nanosight LM10/LM14 system (NanoSight Ltd., Malvern, UK) (*n* = 6). An automatic injection of the samples for each capture was carried out by a syringe pump, three 60-s videos were taken for each dilution, and the analysis was carried out with Nanosight NTA 3.2 software. The concentration and size distribution were presented as the average concentration per cell ± SEM and mean size ± SEM in nanometer (nm), respectively.

#### Flow cytometry

The hMSC sEVs were adhered to CD63-coated magnetic beads (Thermo Fisher Scientific, MA, USA) and analyzed for the presence of the tetraspanins CD9, CD63, and CD81 by flow cytometry according to the manufacturer’s protocol with some small modifications. Briefly, for each sample, 10 μg of sEVs was incubated with 100,000 beads overnight with gentle agitation. The bead-EVs were washed with 0.5% BSA in PBS, incubated with CD9-APC-Vio-770, CD63-PE-Vio-770, and CD81-PerCP-Vio700 or corresponding isotype control (Miltenyi Biotec, Bergisch Gladbach, Germany) for 40 min with gentle agitation at room temperature. The bead-EVs were washed three times and acquired on a FACSVerse (BD Bioscience, San Jose, CA, USA). The data was analyzed using FlowJo™10 (Flow Jo, LLC, Ashland, OR, USA).

#### Transmission electron microscopy

EV analysis by negative staining was performed as previously described [15]. Ten micrograms of vesicles was placed onto glow discharged 200-mesh formvar/carbon copper grids (Electron Microscopy Sciences, Hatfield Township, USA). After two washes in H_2_O, sEVs were fixed in 2.5% glutaraldehyde. After two further washes in H_2_O, the samples were stained with 2% uranyl acetate for 1.5 min. Samples were examined on a digitized LEO 912AB Omega electron microscope (Carl Zeiss SMT, Oberkochen, Germany) at 120 kV with a Veleta CCD camera (Olympus-SiS, Münster, Germany).

#### Protein isolation and Western blot analysis

Proteins from hMSCs were isolated using RIPA buffer (Thermo Fisher Scientific, Gothenburg, Sweden) and protein inhibitors (cOmplete™, Mini Protease Inhibitor Cocktail, Roche, Basal, Switzerland). Small EVs isolated from hMSCs were mixed with RIPA buffer and used for the Western blot analysis. The protein concentrations were measured by Qubit (Thermo Fisher Scientific) according to the manufacturer’s protocol. For Western blot analysis, 20 μg of proteins was loaded, separated on precast 4–20% polyacrylamide Mini-PROTEAN TGX gels (Bio-Rad Laboratories, Hercules, CA, USA), and transferred to PVDF membranes (Bio-Rad Laboratories, Hercules, CA, USA). The membranes were blocked with EveryBlot Blocking Buffer (Bio-Rad Laboratories, Hercules, CA, USA) and incubated with primary antibodies at 4 °C overnight. All primary antibodies were diluted in EveryBlot Blocking Buffer (Bio-Rad Laboratories). The primary antibodies used were Grp94 (1:1000 dilution, clone 9G10, Enzo Life Sciences, Solna, Sweden), anti-CD63 (1:1000 dilution, clone H5C6, BD Biosciences), anti-flotillin-1 (1:1000 dilution, clone EPR6041, Abcam, Cambridge, UK), and anti-Tom20 (1:200 dilution, clone sc-136211, Santa Cruz Biotechnology, Santa Cruz, CA, USA). To investigate the CD63 expression, the separation was performed under non-reducing conditions. For the other proteins, the separation was performed under reducing conditions. The membranes were washed three times in 1× Tris-buffered saline-Tween (TBST) (Bio-Rad Laboratories) before incubation with the secondary antibody for 1 h. The secondary antibody used for anti-flotillin-1 was anti-rabbit IgG (horseradish peroxidase conjugated, 1:5000 dilution, Harlan Sera-Lab, Loughborough, UK), the secondary antibody used for anti-CD63 and anti-Tom20 was anti-mouse IgG (horseradish peroxidase conjugated, 1:5000 dilution, Harlan Sera-Lab), and the secondary antibody used for anti-Grp94 was anti-rat IgG (horseradish peroxidase conjugated, 1:5000 dilution, Harlan Sera-Lab). All the secondary antibodies were diluted in EveryBlot Blocking Buffer (Bio-Rad Laboratories). After incubation with secondary antibodies, the membranes were washed three times in TBST and developed using the SuperSignal West Femto maximum sensitivity substrate (Thermo Fisher Scientific) and a ChemiDoc Imaging System (Bio-Rad Laboratories).

### 3D pellet culture and sEV treatment

Approximately 200,000 DCs were placed in a polypropylene conical tube (Corning Inc., USA) with 0.5 ml of chondrogenic media (DMEM-high glucose added with insulin, transferrin, and selenium (ITS-G; Thermo Fisher Scientific, MA, USA)), 5 mg/ml linoleic acid, 10 ng/ml transforming growth factor beta (TGF-β1; R&D Systems, MN, USA), 14 mg/ml ascorbic acid, 10^− 7^ M dexamethasone (Sigma-Aldrich, MS, USA), 1.0 mg/ml human serum albumin (Equitech-Bio Inc., KV, USA), and 1% penicillin/streptomycin). The cells were centrifuged (470×*g* at 4 °C for 5 min) and incubated (37 °C and 5% CO_2_) for 3–4 h to allow spheroid formation. For the EV treatment group, the media were replaced with 500 μl of chondrogenic media containing sEVs (5 × 10^10^ vesicles/ml). Chondrogenic media without the sEVs served as control. The media were replaced with fresh media with or without sEVs every 48 h, the used media were collected and centrifuged (300×*g*, 4 °C, 5 min), and the supernatants were stored at − 80 °C for further analysis. The pellets were then harvested at days 7, 14, and 28. Four replicates of pellets were cultured for each group and repeated with different donor cells (*n* = 5) separately. Two replicates were used for histology and two for biochemical analysis.

### Cell viability and LDH activity measurement

#### Cell viability

To each pellet, 50 μl of cell counting kit 8 (CCK-8) solution (Dojindo, Munich, Germany) was added and incubated for 4 h (37 °C and 5% CO_2_). The supernatant (100 μl) was collected from each pellet in duplicates, and absorbance was measured at 450 nm by a microplate reader (BioTek, VT, USA). CCK-8 was performed at days 7, 14, and 28 before harvesting of the pellets.

#### LDH activity

Analysis of lactate dehydrogenase (LDH) was done on media collected from the different groups of DC cultures using lactate dehydrogenase (LDH) assay kit (Abcam) following the manufacturer’s instruction. Absorbance was measured at 450 nm by a microplate reader (BioTek, VT, USA). The analysis was performed at days 4, 7, 13, 22, and 28. Technical duplicates were included in all the analyses and repeated with different donor cells (*n* = 5).

### Glycosaminoglycan and DNA assays

DC pellets were solubilized with papain (1.5 mg papain/ml (Sigma-Aldrich, MS, USA), 20 mM sodium phosphate buffer, 1 mM EDTA, and 2 mM dithiothreitol) and incubated at 60 °C overnight. The digested pellets were analyzed by glycosaminoglycan (GAG) and DNA assay kits (Chondrex, WA, USA). The assays were performed according to the manufacturer’s instruction. Absorbance was read at 525 nm for GAG concentration, and fluorescence was read at excitation 360 nm/emission 460 nm for DNA. Analyses were run in duplicates, and data presented as GAG content normalized to total DNA for each pellet.

### TUNEL assay

Histology sections of DC pellets were deparaffinized, and TUNEL assay was carried out according to the manufacturer’s guide (FragEL DNA Fragmentation Detection Kit, Fluorescent-TdT Enzyme, Merck Chemicals and Life Science AB, Sweden). Positive control was generated by submerging the section in 1 μg/μl DNase for 20 min prior to the other steps, and negative control was carried out by keeping the specimen in 1× buffer rather than in reaction mixture. Evaluation was performed with fluorescence microscopy (NIKON Eclipse E600, Japan), and NIS Elements software (Nikon Metrology NV, Europe) was used to perform fluorescent intensity quantification and determine the area of the pellets. The level of apoptosis was presented as pixels per square micrometer.

### Histological staining

Harvested DC pellets were fixed with 4% formaldehyde (Histolab, Gothenburg, Sweden) and sectioned and stained with Alcian blue van Gieson, and the ECM components (proteoglycan and collagen) were evaluated under light microscopy (Nikon Eclipse E600).

### Immunohistochemistry

#### PCNA, SOX9, KRT19, ACAN, COLIIA1

Immunohistochemistry (IHC) was carried out to verify proliferation and characteristics of chondrocyte-like cells in the DCs. Briefly, paraffin-embedded sections were deparaffinized and rehydrated and antigen retrieval (Citrate buffer, pH − 6, 90 °C for 20 min) was carried out. Primary antibodies used include anti-PCNA (1:100, Abcam, Cambridge, USA), anti-Sox9 (1:1000, Abcam, Cambridge, USA), anti-KRT19 (1:100, Abcam, Cambridge, USA), anti-ACAN (1:500, Abcam, Cambridge, USA), and anti-COLIIA1 (1:100, Abcam, Cambridge, USA). After incubation at 4 °C overnight, blocking solution (0.1% Triton X-100, 2% BSA, and 100 mM glycine in PBS) was added; for COLIIA1 sections, blocking with 3% BSA was used. Secondary antibodies include donkey anti-rabbit IgG Alexa Fluor 546 (1:200, Thermo Fisher Scientific, MA, USA) against SOX9, KRT19, PCNA, and ACAN, and goat anti-rabbit IgG Alexa Fluor 546 (1:200, Thermo Fisher Scientific, MA, USA) against COLIIA1. To enhance the detection of COLIIA1, the sections were incubated with SA-HRP (1:100, TSA Plus Cyanine 3 System kit, PerkinElmer, MA, USA) according to the manufacturer’s guideline prior to nuclei counterstaining with ProLong® Gold Antifade Mountant (DAPI; Thermo Fisher Scientific, MA, USA). The samples were examined under fluorescence microscope (Nikon Eclipse E600, Japan), and NIS Elements software (Nikon Metrology NV, Europe) was used to determine the cross-sectional area of the pellets and the number of positive cells and to quantify fluorescence intensity. The level of PCNA, SOX9, ACAN, and COLIIA1 expressions was presented as pixels/square micrometer, while for KRT19, 200 cells were counted and the immunopositive cells were presented as a percentage of the total count. The analysis of the images was conducted in duplicates for each group of pellet and was repeated with different donor cells (*n* = 5).

### Cell media/supernatant analysis

#### Cytokine secretion

Cytokine analysis was performed on cell-free supernatant collected from the DC pellet cultures (EV-treated or control, days 4, 7, 14, 22, and 28). The concentration of cytokines IL-1β, IFN-α2, IFN-γ, TNF, CCL2, IL-6, CXCL8, IL-10, IL-12p70, IL-17A, IL-18, IL-23, and IL-33 was measured using a bead-based immunoassay (LEGENDplex™; Human Inflammation Panel 1, BioLegend, San Diego, USA) in accordance with the manufacturer’s instructions. The samples were acquired on a FACS Verse (BD Bioscience, San Jose, CA) running FACSuite software (BD Bioscience). Data analysis was performed on FCAP Array software (Soft Flow Ltd., Pécs, Hungary).

#### MMP-1 secretion

The supernatant from pellet media was analyzed using human MMP1 ELISA Kit (Abcam, Cambridge, UK) following the manufacturer’s instructions. Absorbance was measured at 450 nm by a microplate reader (BioTek, VT, USA).

All supernatant analyses were conducted with technical duplicates and were repeated with different donor cells (*n* = 5).

### Statistical analysis

All statistical data are presented as mean ± SEM. Data were analyzed via SPSS 25.0 software (IBM SPSS Statistics, NY, USA). Two-tailed Student’s *t* test was used to compare the means between two groups, and multivariate ANOVA with Tukey’s post hoc was used for multiple comparison. *p* < 0.05 was considered as statistically significant.

## Results

### Characterization of hMSCs and sEVs confirmed their characteristics

**Fig. 1:**
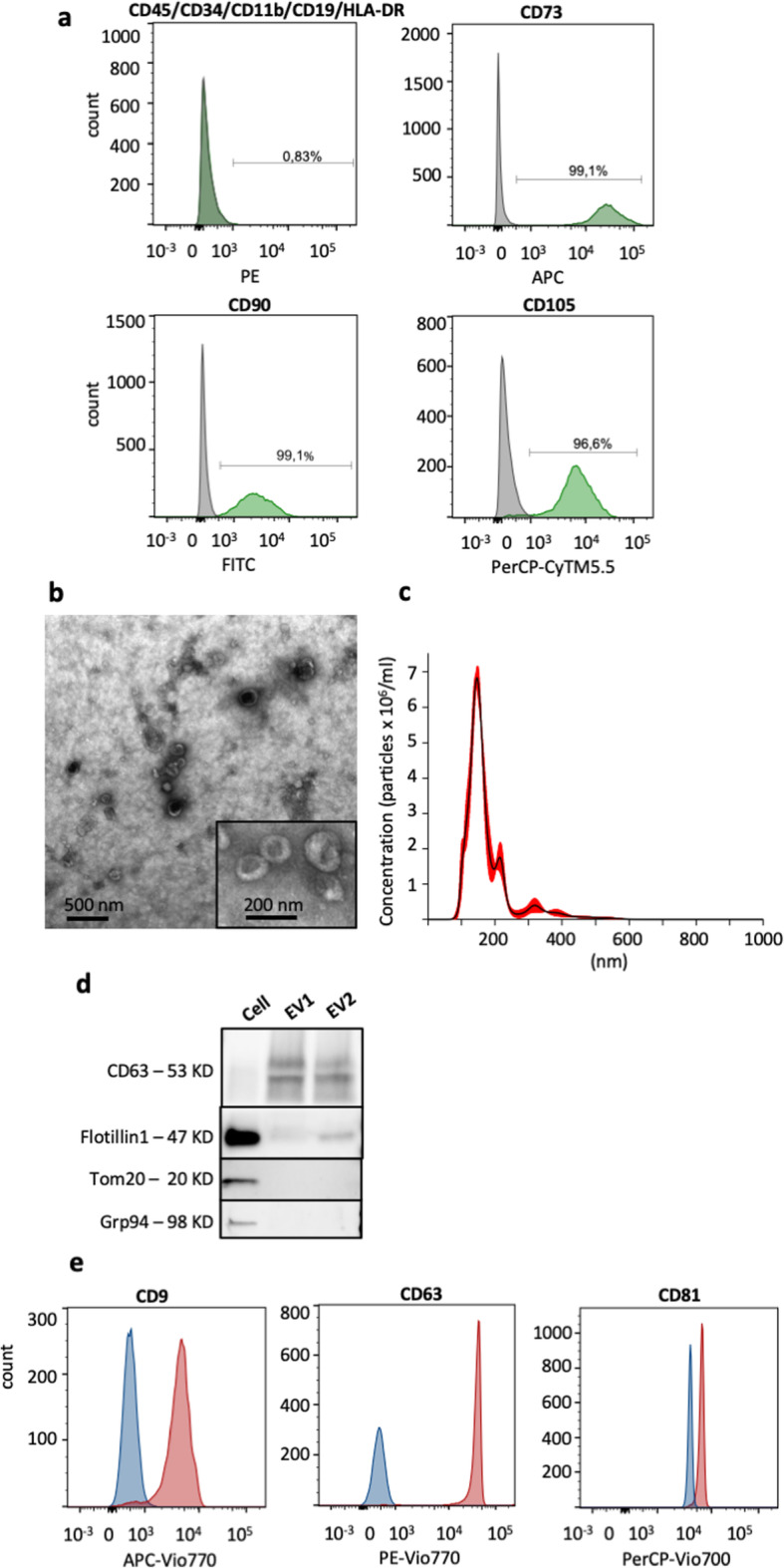
Characterization of hMSC-derived extracellular vesicles (EVs). **a** Bone marrow-derived hMSCs express the common MSC markers CD73, CD90, and CD105 and are negative for the hematopoietic markers CD45, CD340, CD11b, CD19, and HLA-DR (green curve, antibody stained sample; gray curve, isotype control). **b** Transmission electron microscopy (TEM) of sEVs. **c** Representative nanoparticle tracking analysis histogram shows the size distribution of sEVs. **d** Western blot analysis of two different isolations of hMSCs’ sEVs (EV1 and EV2) and hMSCs (cell) shows that EVs contain the EV markers CD63 and flotillin-1 and are absent from the mitochondrial protein Tom-20 and ER protein Grp94. **e** Flow cytometry of sEVs bound to CD63 beads shows that sEVs are positive for the tetraspanins CD9, CD63, and CD81 (red curve, antibody stained sample; blue curve, isotype control)

Human MSCs were isolated and expanded from bone marrow aspirates and further characterized using flow cytometry to confirm the mesenchymal lineage. The surface markers CD73, CD90, and CD105 of hMSCs were detected, and the hematopoietic lineage markers CD45, CD34, CD11b, CD19, and HLA-DR were absent (Fig. 1a) confirming the phenotypical characteristic of the isolated hMSCs. Extracellular vesicles (EVs) were isolated from the hMSC conditioned media (CM) using ultracentrifugation and analyzed by transmission electron microscopy (TEM), nanoparticle tracking analysis (NTA), flow cytometry, and Western blot to evaluate the integrity, size, concentration, and presence of EV markers (Fig. 1b–e). TEM pictures show cup-shaped elements, typical vesicle-like structures, with the size between 50 and 150 nm (Fig. 1b). NTA revealed that the size of sEVs ranges between 100 and 250 nm with the mean and mode size of 175 ± 5.79 nm and 144 ± 2.22 nm, respectively (Fig. 1c). The number of sEVs secreted per hMSC was quantified to be 3.2 ± 0.38 × 10^5^. Western blot analysis showed that isolation of sEVs at two different batches, EV1 and EV2, expressed the typical exosome markers CD63 and flotillin-1 (Fig. 1d). The endoplasmic reticulum (ER) protein Grp94 and mitochondrial protein Tom20 were only expressed in hMSC cellular proteins and not in the EVs, indicating no contamination of ER and mitochondria in the isolated EVs. Flow cytometry of EVs bound to CD63 beads shows that the tetraspanins CD9, CD63, and CD81 are detected on the membrane of the EVs (Fig. 1e). Collectively, these results indicate that we mainly isolated small EVs (sEVs < 200 nm), with the characteristics of exosomes.

### Small EVs promoted cell proliferation/viability and suppressed apoptosis in DCs

**Fig. 2:**
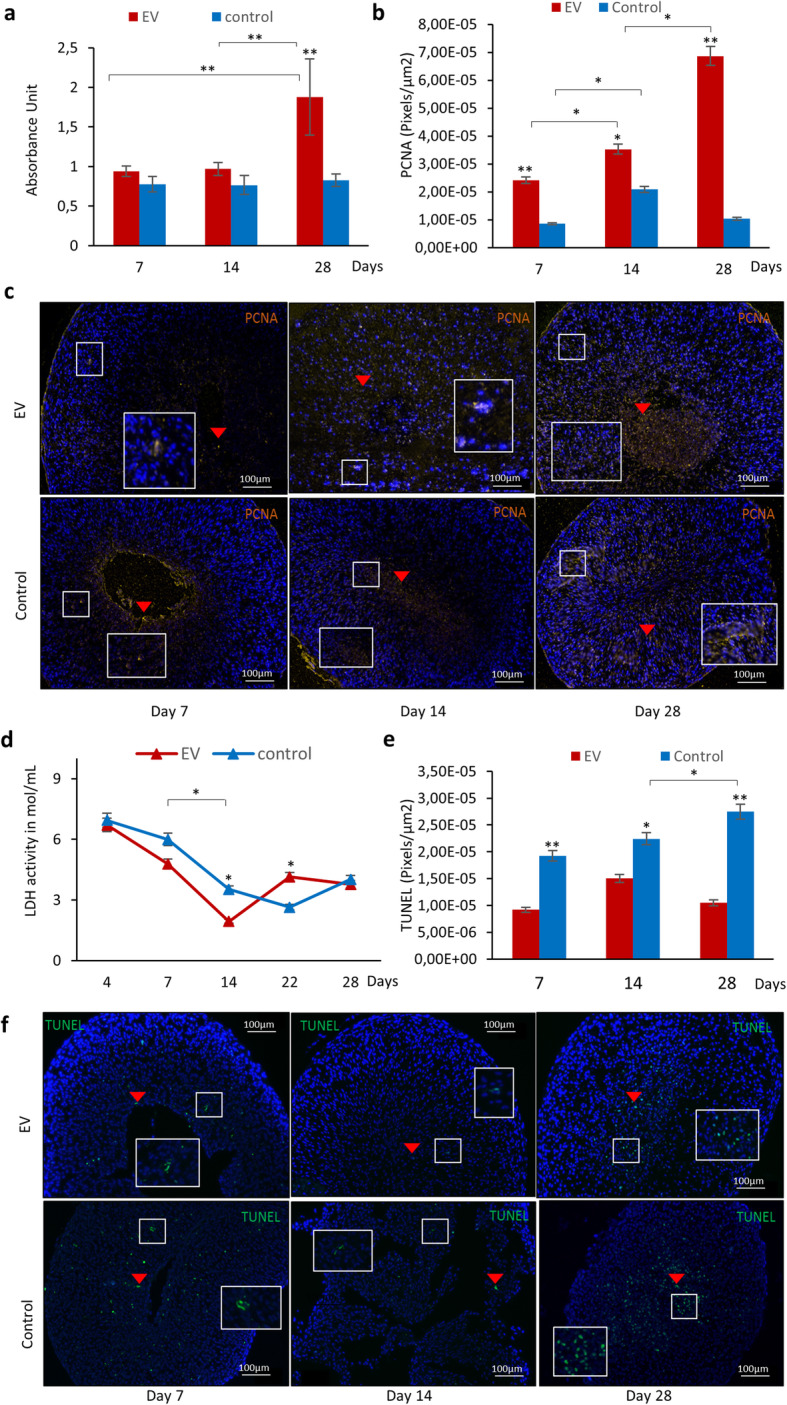
Cell proliferation and expression of cell proliferation markers after EV treatment. **a** Illustration of cell proliferation from day 7 to 28 in DC pellets treated with EVs compared to control. The result is presented in the unit of absorbance. **b** The bar graph presents the quantitative level of PCNA-positive cells in histological sections of DCs treated with sEVs compared to control from day 7 to 28. The results are presented as pixels/square micrometer. **c** Representative images of the expressions of PCNA in DC pellets treated with sEVs from day 7 to 28. Yellow colors indicated by red arrows are the positive expressions of PCNA, while green colors are that of apoptotic cells in the pellets. Blue dots are the nuclei of DCs. **d** LDH activity of DC pellets. The line graph illustrates LDH activity (cell death) in DC pellets from day 7 to 28. **e** The bar graph presents the quantitative level of TUNEL assay in histological sections of DCs treated with sEVs compared to control from day 7 to 28. **f** Representative images of the expressions of apoptotic cells in DC pellets treated with sEVs from day 7 to 28, respectively. Values are the mean ± SEM (*n* = 5).*p < 0.05, **p < 0.01

In order to explore how hMSC-derived sEVs affect DCs’ proliferation, CCK-8 assay and immunohistochemistry for PCNA were carried out reflecting the viable cell number as well as the presence of proliferating cells in the DC pellets. The result from CCK-8 revealed that DC pellets stimulated with sEVs showed a continuous cell proliferation from day 7 to day 28 with significantly higher number of viable cells (*p* < 0.01) compared to control at day 28. The number of viable cells further doubled from day 14 to day 28 in the EV-stimulated group, while that of the control group remained the same (Fig. 2a). PCNA expression was detected in both EV-treated and non-treated pellets; however, in the EV group, a significantly higher level was exhibited compared to control (Fig. 2b, c), and the level was observed to follow a similar pattern to that of CCK-8 assay confirming increased cell proliferation with sEV stimulation.

LDH assay was used as an indicator of cell death, and TUNEL assay was performed additionally to investigate apoptosis in DC pellets. Lower LDH activity was detected in pellets treated with sEVs compared to control from day 4 to day 14 indicating low cell death with sEV treatment; however, the level raised at day 22 and declined slightly at day 28 (Fig. 2d). TUNEL assay revealed the presence of cells with fragmented DNA in both groups (Fig. 2e, f); however, at all time points, significantly lower level was detected in sEV-treated pellets compared to control with an increasing level observed from day 7 to 28 in the control group. This result conforms to that of LDH activity validating that stimulation with sEVs suppressed cell death in DC pellets.

### Small EVs induced early chondrogenesis in DCs from degenerated IVDs

**Fig. 3:**
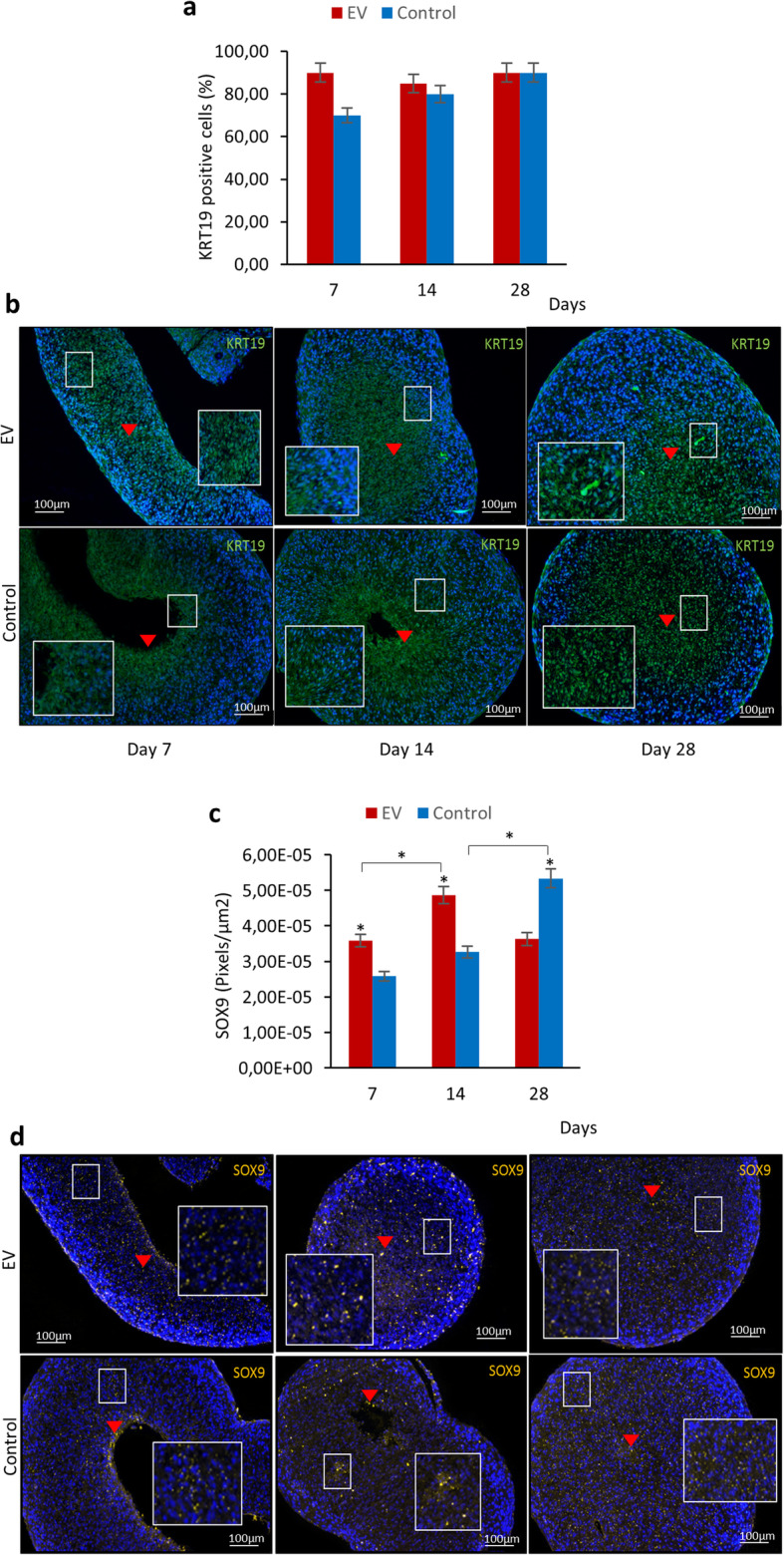
Chondrocyte-like cell characteristics of DCs isolated from degenerated IVDs after hMSCs’ sEV treatment. **a** The bar graph presents the percentage of KRT19-positive cells in histological sections of DCs treated with sEVs compared to control from day 7 to 28. **b** Representative images showing the expressions of KRT19 in DC pellets treated with sEVs from day 7 to 28. **c** The bar graph illustrates the quantitative level of SOX9-positive cells in histological sections of DC pellets for the same time points. **d** Representative images showing the expression of SOX9 in DC pellets treated with sEVs from day 7 to 28. Quantitative results in **a** and **c** are presented as pixels/square micrometer while green colors in **b** and **d** indicated by red arrows are the positive expressions of KRT19, while yellow colors are that of SOX9 in the pellets, respectively. Blue dots are the nuclei of DCs. Data represents mean ± SEM (*n* = 5). *p < 0.05

To validate the characteristic of NP/chondrocyte-like cells in DCs isolated and to confirm the occurrence of chondrogenesis, the presence of KRT19 and SOX9 was investigated by IHC as KRT19 is one of the markers of NP cells [16] and SOX9 is known as the early indicator of chondrogenesis [17]. The expression of KRT19 was observed in all the pellets confirming the chondrocyte-like cell characteristics of the DCs isolated (Fig. 3a, b). SOX9 expression was detected throughout the time points; however, significantly higher level was detected in pellets stimulated with sEVs at days 7 and 14 compared to control (Fig. 3c, d). At the latest time point, 28 days, SOX9 expression was increased in the control compared to the EV-treated group. This implied that chondrogenesis took place earlier when treated with sEVs compared to control.

### Small EVs enhanced early production of ECM

To reverse IVD degeneration, it is vital to influence the DCs in degenerated IVDs to become viable and to produce ECM in order to preserve the integrity of the discs. In an attempt to investigate if the sEVs from hMSCs could influence the DCs to produce vital ECM, GAG assay, Alcian Blue van Gieson (ABvG) histology staining, and IHC were carried out to investigate the production of GAG, collagen type II (COLIIA1), and aggrecan (ACAN) in DC pellets from day 7 to 28.

**Fig. 4:**
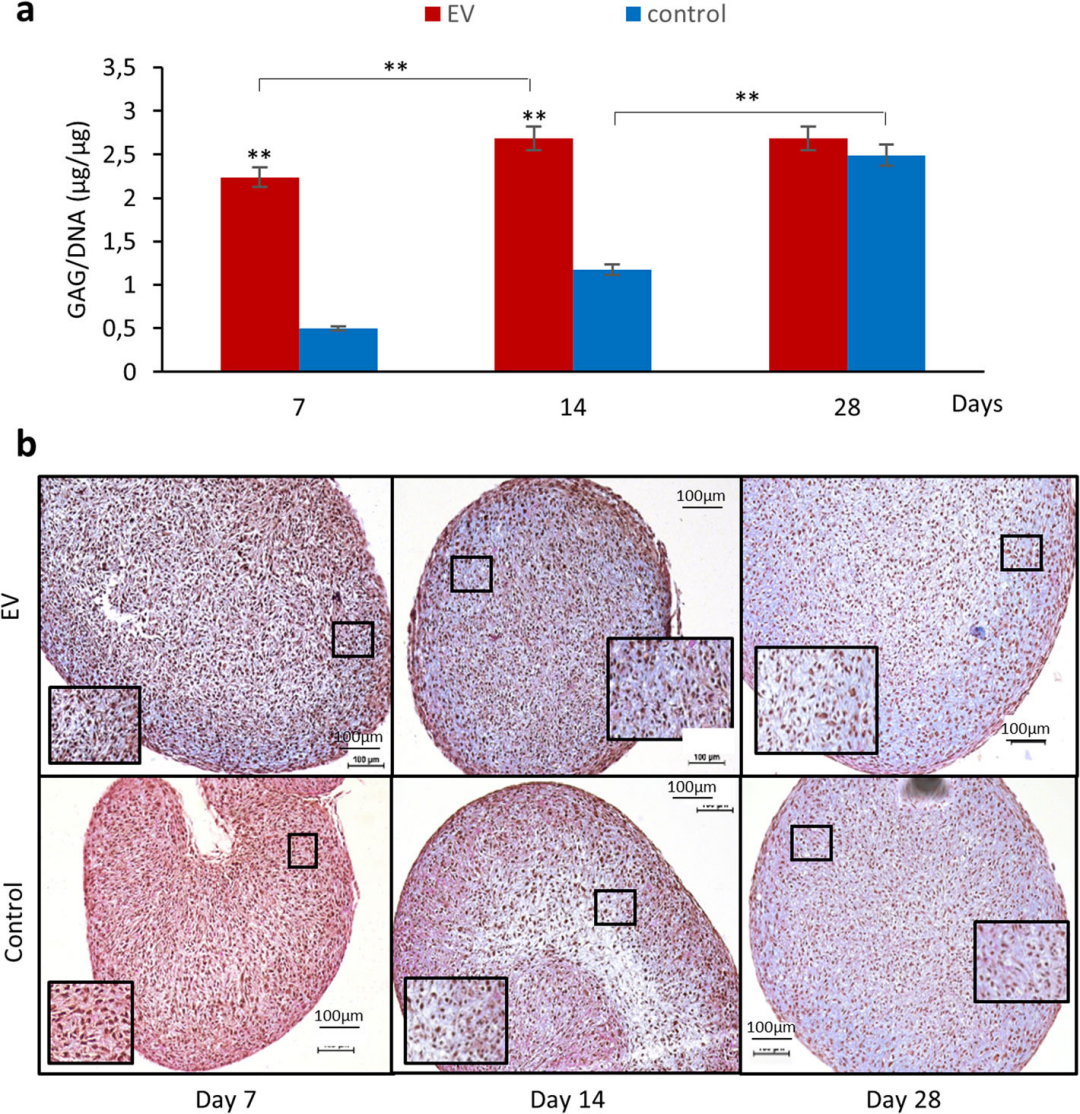
GAG production and histological staining of DC pellets treated with sEVs from day 7 to 28. **a** Quantification of GAG production in DC pellets. Values presented in bar charts are the mean ± SEM (*n* = 5).*p < 0.05 and **p < 0.01. **b** Alcian Blue van Gieson staining of DC pellets. Histology sections present proteoglycan (blue) and collagen (pink), and black dots represent nuclei of the cells

GAG assay showed an early production of GAG after EV treatment, with significantly higher levels in DCs treated with sEVs compared to control at days 7 and 14 (*p* < 0.01). However, a continuous increase in GAG production was observed in the control group from day 7 to 28, while in the EV group, a significantly high production was observed from day 7 to day 14 where the pellets yielded highest production already at day 14 (Fig. 4a). This is supported by the results from ABvG staining as DCs treated with sEVs showed high proteoglycan accumulation as early as in day 7 and throughout the culture period (Fig. 4b), confirming that sEVs from hMSCs promoted an increased and early production of ECM in degenerated DCs.

**Fig. 5:**
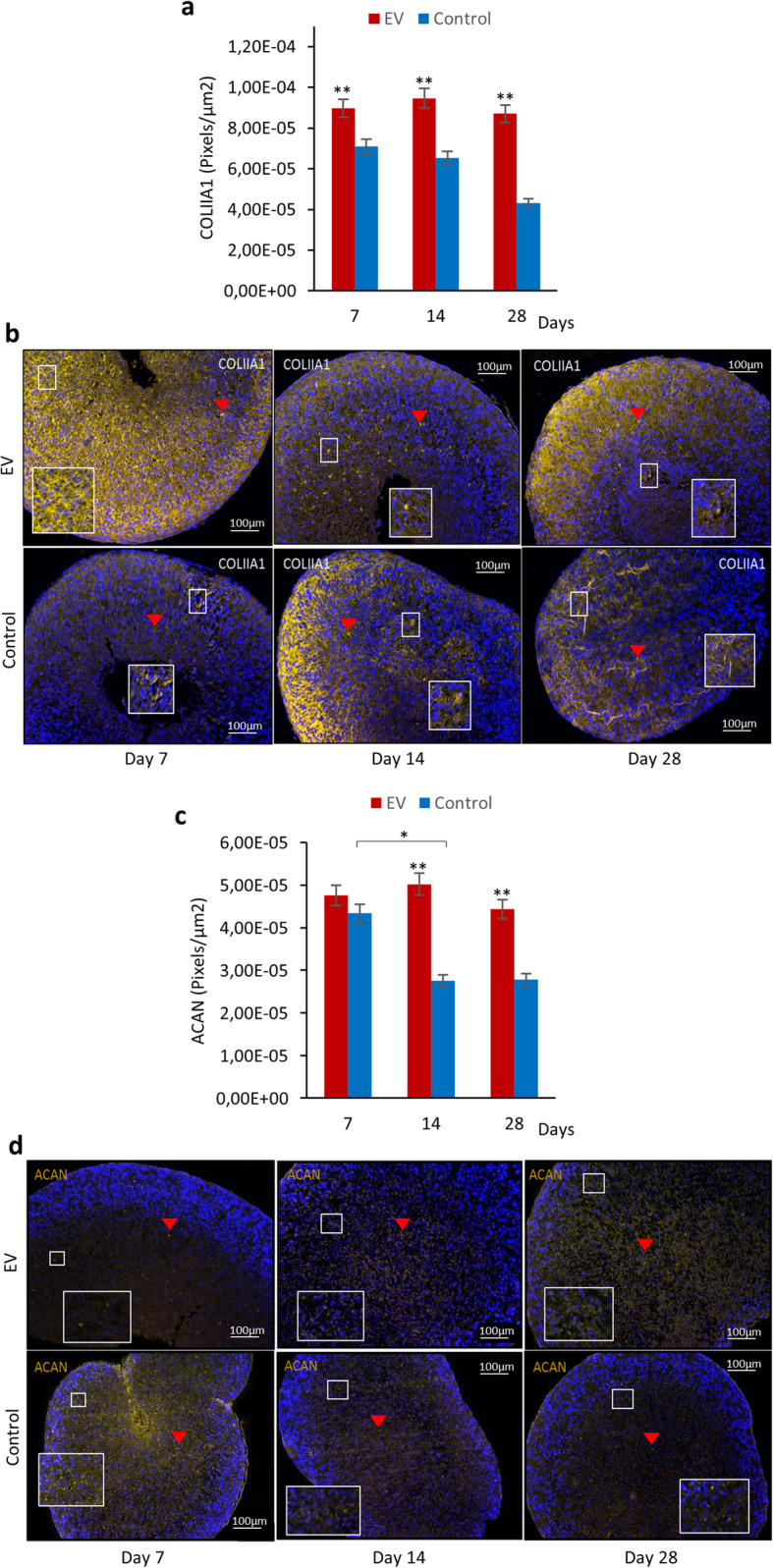
Expressions of collagen type II (COLIIA1) and aggrecan (ACAN) in DC pellets over a 28-day period. **a** The bar graphs illustrate the level of COLIIA1-positive cells in DC pellets. **b** Representative images showing the expressions of COLIIA1 in DC pellets treated with sEVs from day 7 to 28. **c** The bar graphs illustrate the level of ACAN-positive cells in DC pellets. **d** Representative images showing the expressions of ACAN in DC pellets treated with sEVs from day 7 to 28. Quantitative results in **a** and **c** are presented as pixels/square micrometer while yellow colors indicated by red arrows in **b** and **d** are positive expressions of the proteins in the pellets. Blue dots are the nuclei of DCs. Data represents mean ± SEM (*n* = 5). *p < 0.05 and **p < 0.01

COLIIA1 and ACAN are some of the main ECM compositions of the nucleus pulposus; in fact, COLIIA1 is also known as the late indicator of chondrogenesis [18, 19]. In DC pellets treated with sEVs, high COLIIA1 expression was observed as early as in day 7 and the level was significantly higher than control and remained similar throughout the experiment (Fig. 5a, b). The level of COLIIA1 and ACAN remained at the same expression level throughout the experiment in the EV group. Furthermore, the level of ACAN expression after sEV treatment was significantly higher than control in days 14 and 28 (Fig. 5c, d). This finding is consistent with that of GAG assay, and it gives affirmation that sEV treatment expedited and enhanced ECM production in DC pellets.

### Secreted cytokines and MMP-1 levels in cell media

**Fig. 6:**
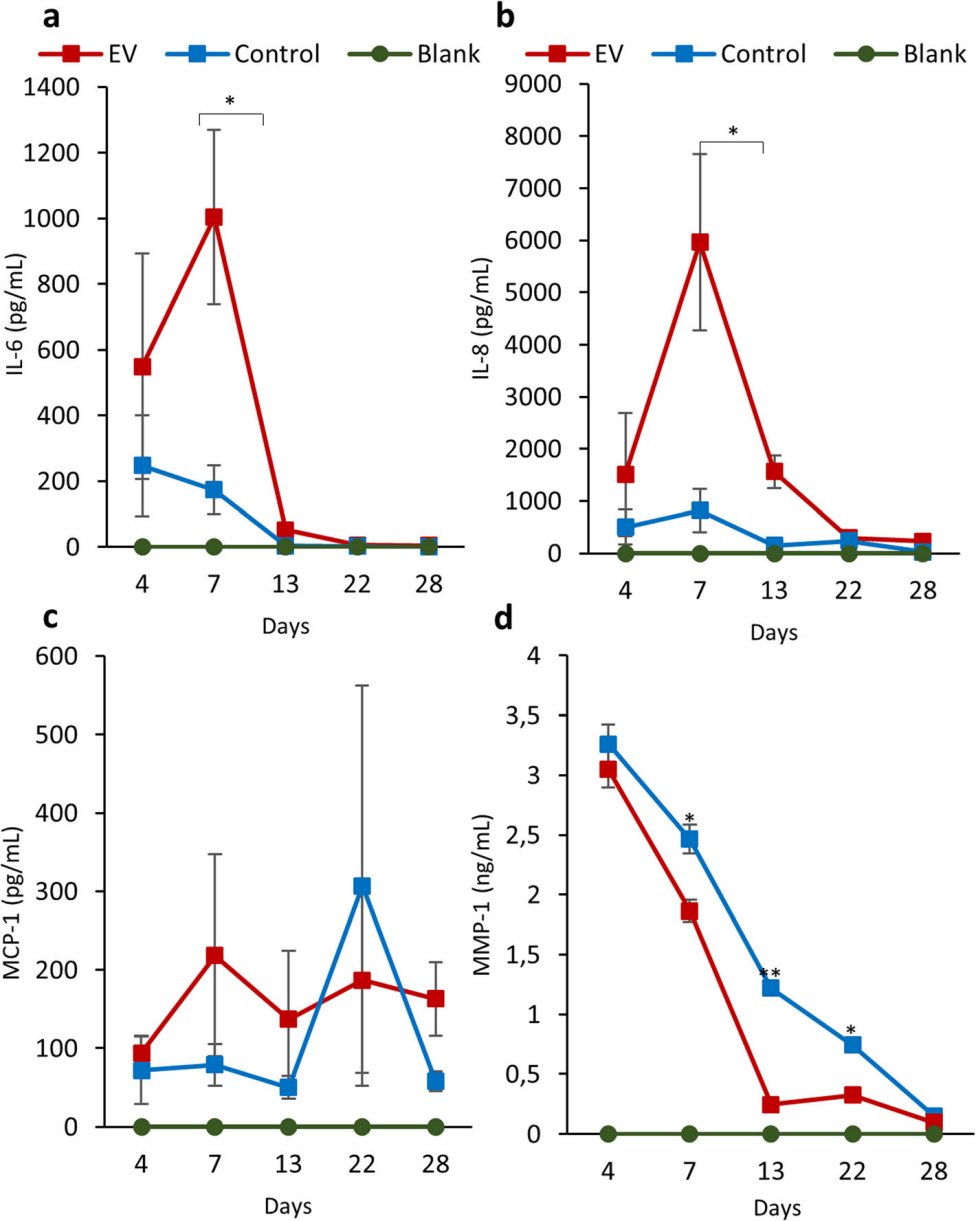
The secretion of cytokines and MMP-1 by the DCs after sEV treatment. **a–c** The line graphs illustrate the concentration of cytokines IL-6, IL-8, and MCP-1 respectively, secreted by the DCs over a 28-day period. **d** The line graph represents the level of MMP-1 secreted by DCs treated with sEVs compared to control. Data represents mean ± SEM (*n* = 5). *p < 0.05. Fresh media without the sEVs served as blanks

In order to study the secretory response of DCs after exposure to the hMSCs’ sEVs, the concentration of different cytokines (IL-1β, IFN-α2, IFN-γ, TNF, CCL2, IL-6, CXCL8, IL-10, IL-12p70, IL-17A, IL-18, IL-23, and IL-33) and MMP-1 was measured. However, only monocyte chemoattractant protein 1 (MCP-1), interleukin 6 (IL-6), and interleukin 8 (IL-8) were detected in the media. The level of IL-6 and IL-8 in the medium from EV-treated pellets was highest at day 7 and decreased at later time points. However, no significant differences between EV-treated pellets and control were detected for any of the cytokines (Fig. 6a–c). The release of MMP-1, which is a collagenase responsible for degradation of collagens in the IVDs during IVD degeneration [20], decreased over time in both EV-treated and control (Fig. 6d). Interestingly, DCs treated with hMSCs’ sEVs released a lower level of MMP-1 compared to control.

## Discussion

Cell therapy approaches such as injection of MSCs have been recognized as a promising treatment strategy for LBP caused by IVD degeneration since MSCs can differentiate into chondrocyte-like cells and promote cell proliferation [21] and inhibit apoptosis [13]. However, several studies have provided compelling evidence indicating that the therapeutic effects of MSCs are mainly mediated by paracrine mechanisms, particularly the secretion of EVs [10, 12, 22, 23]. Therefore, the use of hMSC-derived EVs may serve as a more appropriate alternative for treatment of IVD degeneration. Unlike hMSCs, EVs are more stable and not easily influenced by the inflammatory microenvironment in the degenerated IVDs [24]. EVs may therefore be used to promote IVD repair by combating destructive effects of pro-inflammatory cytokines and MMPs in the disc microenvironment.

In this study, it is demonstrated in a 3D in vitro model that hMSC-derived sEVs may fulfill these requirements. The results revealed that sEVs can improve cell viability and proliferation and expedite chondrogenesis in DCs from degenerated IVDs of patients suffering severe LBP. EV treatment induced early production of crucial ECM components such as proteoglycan, aggrecan, and collagen type II while suppressing apoptosis and the secretion of MMP-1, a process that is essential for proper IVD regeneration. Small EVs also increased viability and proliferation of DCs.

The level of proliferation of DCs in pellet culture over a 28-day period was previously reported to be consistent throughout the culture period and therefore speculated that it was because these DCs were mature cells exposed to degenerative environment and were hard to influence [8]. However, with sEV treatment, a continuous increase in the level of proliferating cells was detected from day 7 to 28, which is in line with previous report where exosomes promoted cell proliferation of nucleus pulposus (NP) cells through cellular uptake of the vesicles [25]. These findings show that proliferation of mature DCs can be promoted given the right stimulus.

Previous studies have reported that exosomes isolated from MSCs inhibit apoptosis in cartilage cells [11, 13] and ameliorate endoplasmic reticulum stress-induced apoptosis by activating AKT and ERK signaling in a rat-tail model [26]. In this study, reduced LDH activity and apoptosis were observed in DC pellets stimulated with sEVs indicating that sEVs could be the key bioactive factors for preventing DC’s apoptosis in the degenerated IVDs.

The presence of SOX9 and KRT19 expressions [18, 19] also confirmed the chondrocyte-like cell characteristic of the DCs isolated. Another characteristic of chondrocyte-like cells is the ability to produce ECM [27]. The main compositions of IVDs are collagen and proteoglycans forming a strong framework that holds the cells in the ECM where matrix degrading enzymes (MMPs) and their inhibitors are kept in homeostasis. As IVD degeneration progresses, the concentration of pro-inflammatory factors along with MMPs increases while the concentration of MMP inhibitors decreases, resulting in an increased matrix degradation [27, 28]. This threatens the microenvironment of the cells as they depend on the ECM for survival [2]. In DCs from degenerated IVDs, stimulation with sEVs induced an earlier and a significantly higher proteoglycan accumulation, GAG production, and expressions of COLIIA1 and ACAN compared to the control. This increased production of ECM may account for the suppressed MMP-1 production by the DCs as the ECM provided the DCs with favorable environment. Exosomes derived from hMSCs were also reported to suppress H_2_O_2_-induced NP cell inflammation [29]. This means that sEVs were able to assist the DCs to rebalance homeostasis in this 3D model. This was further supported by the decreased concentration of MMP-1 in the media in the EV group.

Previous findings report promising results when stimulating DCs with growth factors such TGF-beta and BMP-3 and also with conditioned media derived from hMSCs [8, 30, 31] as these signaling molecules are believed to act either on themselves (autocrine) or on neighboring cells (paracrine) [32, 33] to bring about the observed regenerative effects. In this study, it was demonstrated that with EV stimulation, an enhanced ECM production was observed as early as in day 7 in these 3D pellet culture models. It is of interest to understand which molecules are responsible for these effects; however, in this study, the content of the hMSCs’ sEVs was not analyzed in depth. On the other hand, small interdonor variation was detected, and this could be due to the fact that the disc tissues used in the study were at similar levels of degeneration. It could also be speculated that the concentration of EVs used in this study had a strong impact on the degenerated disc cells as this concentration was slightly higher than previously used concentration [34]. The rationale behind using this slightly higher concentration was on the grounds that a 3D model was used for this study and to ensure that enough EVs were present to influence the cells.

EVs from hMSCs have previously been shown to contain a wide range of different growth factors, cytokines, and RNA [35]. It is reasonable to speculate that a mixture of these factors is responsible for causing the effects in the DCs. It could be speculated that the sEVs either were internalized by the DCs [13] for intercellular communication or were delivering bioactive molecules involved in direct cell stimulation [36].

EVs are further known for their involvement in cytokine transportation [37]. Both IL-6 and IL-8 have been found to be associated with secretory vesicles. Upon stimulation with IL-1, mast cells have been reported to release IL-6 containing vesicles [38] while IL-8 has been shown to be transported in the sEVs as inflammatory mediators [39, 40]. The higher levels of IL-6 and IL-8 detected in the media in the EV group compared to control at day 7 are most likely caused by DC secretion after sEV stimulation. However, we cannot rule out that the detected IL-6 and IL-8 are soluble and not bound to the EV membrane.

## Conclusion

Human MSC-derived sEVs may have a potential for therapeutic applications in patients with LBP and disc degeneration, as they mimic several positive biological actions of hMSCs. It may also limit the issues of cell maldifferentiation or mutations and cell handling concerning cell therapy. Thus, the findings from this study open up treatment strategies where sEVs can be engineered to express and deliver molecules that can direct target cells towards regeneration.

## Data Availability

All data generated or analyzed during this study are included in this article.

## Abbreviations

ACAN: Aggrecan
ABvG: Alcian blue van Gieson
BMA: Bone marrow aspirate
COLIIA1: Collagen type II
DCs: Disc cells
DNA: Deoxyribonucleic acid
ECM: Extracellular matrix
FGF: Beta fibroblast growth factor
GAG: Glycosaminoglycan
hMSCs: Human mesenchymal stem cells
HSE: Human serum
IL-6: Interleukin-6
IL-8: Interleukin-8
IVD: Intervertebral disc
LDH: Lactate dehydrogenase
LBP: Low back pain
MSCs: Mesenchymal stem cells
MMP-1: Metalloproteinase-1
NTA: Nanoparticle tracking analysis
NP: Nucleus pulposus
sEVs: Small extracellular vesicles
WB: Western blotting
