# Correction to: Extracellular vesicles from human mesenchymal stem cells expedite chondrogenesis in 3D human degenerative disc cell cultures

**DOI:** 10.1186/s13287-020-01959-2

**Published:** 2020-10-16

**Authors:** Daphne Hingert, Karin Ekström, Jonathan Aldridge, Rossella Crescitelli, Helena Brisby

**Affiliations:** 1 Department of Orthopedics, Institute of Clinical Sciences, Sahlgrenska Academy, University of Gothenburg, Gothenburg, Sweden; 2 Sahlgrenska Cancer Center, Department of Surgery, Institute of Clinical Sciences, Sahlgrenska Academy, University of Gothenburg, Gothenburg, Sweden; 3 Wallenberg Centre for Molecular and Translational Medicine, University of Gothenburg, Gothenburg, Sweden; 4 Department of Rheumatology and Inflammation Research, Institute of Medicine, University of Gothenburg, Gothenburg, Sweden; 5 Department of Orthopedics, Sahlgrenska University Hospital, Gothenburg, Sweden

**Correction to: Stem Cell Res Ther (2020) 11:323**

**https://doi.org/10.1186/s13287-020-01832-2**

The original article [1] contains a typo in co-author, Rossella Crescitelli’s name. The correct presentation is found in this Correction article.
